# Supplementary material for: A Rapid Review Exploring the Role of Yoga in Healing Psychological Trauma
Source: Int J Environ Res Public Health. 2022 Dec 3;19(23):16180. doi: 10.3390/ijerph192316180 (PMC9741324; doi:10.3390/ijerph192316180)
Supplement: Supplementary file 1 [file ijerph-19-16180-s001.zip › ijerph-2045177-supplementary.pdf]

Supplementary Material S1: Sample data extraction form

Bibliographic information

<table>
<tr><td>Study ID:</td><td>Date of extraction:</td><td rowspan="2">Extracted by:</td></tr>
<tr><td>Year published:</td><td>Country:</td></tr>
<tr><td colspan="3">Title:</td></tr>
<tr><td colspan="3">Author(s):</td></tr>
</table>

Study characteristics

Aims/purpose:
Study design:
Study procedures:

Participant characteristics

<table>
<tr><td>Age:</td><td>Gender:</td><td>Sample size:</td></tr>
<tr><td colspan="3">Trauma type:</td></tr>
</table>

Intervention

Intervention type:
Comparator:
Setting:
Duration:

Outcomes

Main themes identified:

Results

Key findings:
Limitations:

Supplementary Material S2: Characteristics of included studies

Reference number	Lead author, publication year	Country	Sample size	Participants' age range (years)	Participants' gender	Yoga intervention, duration, control	Trauma type
23	Braun, 2021	USA	5	27-57	F	• Trauma sensitive yoga (TSY)<br>➢ 1x weekly 75-min class<br>➢ 2 cohorts: 12 weeks and 8 weeks<br>➢ No control	• US veteran women with history of military sexual trauma<br>• Enrolment in current mental health treatment
15	West, 2017	USA	31	18-58	F	• TSY<br>➢ 1x weekly 60-min class<br>➢ 10 weeks<br>• Control: supportive women's health education – not interviewed therefore not included in sample size	• Participants met diagnostic criteria for PTSD using Clinician-Administered PTSD Scale (CAPS)<br>• Trauma occurred at least 12 years prior<br>• Treatment unresponsiveness – at least 3 years of prior PTSD therapy<br>• Currently enrolled in psychotherapy for

							minimum 6 months prior to study
16	Morrison, 2022	South Africa	7	20-26	5F<br>2M	• Kundalini yoga<br>• Purposeful sampling to identify participants who are currently practising yoga and have experienced trauma<br>• Participants had all finished yoga teacher training<br>• No control	• Adverse Childhood Experience Questionnaire (ACE-Q) used to determine extent of exposure to traumatic events
17	Rhodes, 2015	USA	39	18-58	F	• TSY<br>➢ 1x weekly 60-min class<br>➢ 10 weeks<br>• Control: seminar on women's health – not interviewed therefore not included in sample size	• All women met criteria for PTSD as established by CAPS
18	Crews, 2016	USA	8	14-60	F	• Purposeful sampling to identify participants who are currently practising TSY<br>• No control	Survivors of sexual violence

19	Schmid, 2021	USA	67	52.4 mean	20F<br>47M	• TSY<br>➢ 1x weekly 90-min class<br>➢ 15 weeks<br>• Control: wellness lifestyle programme – not interviewed therefore not included in sample size	All participants met criteria for PTSD as established by CAPS
24	LaChiusa, 2016	USA	6	20-59	F	• Purposeful sampling to identify participants who are currently practising Ashtanga yoga and have experienced trauma in the past<br>• The majority of participants (54.8%) report practising 5-6 days per week<br>• Remainder practised 3-4 days per week (41.9%) or 1-2 days per week (3.2%)<br>• Most participants had practised for 1-2 years but some >10 years<br>• No control	At least moderate levels in one of the subscales of abuse or neglect in the Childhood Trauma Questionnaire (CTQ)

20	Cushing, 2018	USA	9 (5 did intervention, 4 did not)	22-52	8M<br>1F	• TSY<br>➢ At least five 60-min weekly yoga sessions in 6 weeks<br>• Control – no yoga	Veterans of post-9/11 conflicts w PTSD symptoms
25	Justice, 2019	USA	4	18+	1F<br>3M	• TSY<br>➢ 90-min classes<br>➢ 10-weeks<br>• No control	Presence of trauma symptoms (as measured by PCL-5)
21	Jindani, 2015	Canada	40	18-63	31F<br>9M	• TSY<br>➢ Weekly 90-min classes<br>➢ 8-weeks<br>➢ 15-min home practice also assigned to participants<br>• Waitlist control group – they were offered KY participation after the 8-week waiting period – not interviewed therefore not included in sample size	PTSD eligibility using PCL-17

22	Gulden, 2016	USA	11	24-62	10F<br>1M	• Retrospective<br>• Participants had to have practised yoga weekly for a minimum of 3 months (style of yoga not specified)<br>• Yoga experience ranged from 1 year to 15 years	Participants experienced interpersonal trauma, as defined by WHO

CAPS: 30-item structured interview designed to make categorical PTSD diagnosis

ACE-Q: 10-item questionnaire used to measure childhood trauma

CTQ: Brief survey of six early traumatic experiences (death, divorce, violence, sexual abuse, illness or other), to assess individual's understanding of their childhood trauma

PCL-5: 20-item self-report measure that assesses the 20 DSM-5 symptoms of PTSD

PCL-17: 17-item self-report measure that assesses 17 of the DSM-5 symptoms of PTSD
